# Structural Analysis of the Complex of Human Transthyretin with 3′,5′-Dichlorophenylanthranilic Acid at 1.5 Å Resolution

**DOI:** 10.3390/molecules27217206

**Published:** 2022-10-25

**Authors:** Vivian Cody, Jia Q. Truong, Bruce A. Holdsworth, Jessica K. Holien, Samantha J. Richardson, David K. Chalmers, David J. Craik

**Affiliations:** 1 Hauptman-Woodward Medical Research Institute, Inc., 700 Ellicot St., Buffalo, NY 14203, USA; 2 School of Science, RMIT University, Bundoora, VIC 3083, Australia; 3 Monash Institute of Pharmaceutical Sciences, Monash University, Parkville, VIC 3052, Australia; 4 Institute for Molecular Bioscience and Australian Research Council Centre of Excellence for Innovations in Peptide and Protein Science, The University of Queensland, Brisbane, QLD 4072, Australia

**Keywords:** human transthyretin, TTR, binding affinity, molecular structure, halogen bonding

## Abstract

Human transthyretin (hTTR) can form amyloid deposits that accumulate in nerves and organs, disrupting cellular function. Molecules such as tafamidis that bind to and stabilize the TTR tetramer can reduce such amyloid formation. Here, we studied the interaction of VCP-6 (2-((3,5-dichlorophenyl)amino)benzoic acid) with hTTR. VCP-6 binds to hTTR with 5 times the affinity of the cognate ligand, thyroxine (T_4_). The structure of the hTTR:VCP-6 complex was determined by X-ray crystallography at 1.52 Å resolution. VCP-6 binds deeper in the binding channel than T_4_ with the 3′,5′-dichlorophenyl ring binding in the ‘forward’ mode towards the channel centre. The dichlorophenyl ring lies along the 2-fold axis coincident with the channel centre, while the 2-carboxylatephenylamine ring of VCP-6 is symmetrically displaced from the 2-fold axis, allowing the 2-carboxylate group to form a tight intermolecular hydrogen bond with Nζ of Lys15 and an intramolecular hydrogen bond with the amine of VCP-6, stabilizing its conformation and explaining the greater affinity of VCP-6 compared to T_4_. This arrangement maintains optimal halogen bonding interactions in the binding sites, via chlorine atoms rather than iodine of the thyroid hormone, thereby explaining why the dichloro substitution pattern is a stronger binder than either the diiodo or dibromo analogues.

## 1. Introduction

Transthyretin (TTR) is one of three plasma proteins that is responsible for the distribution of thyroid hormones (THs) in the blood of vertebrates. The two main thyroid hormones are tetraiodo-L-thyronine (thyroxine; T_4_) and triiodo-L-thyronine (T_3_) [1]. Human TTR can cause two types of diseases due to misfolding, both of which belong to the amyloidosis family of diseases. Familial Amyloidotic Polyneuropathy (FAP) is an autosomal dominant inherited disease that can be caused by one of more than 100 point mutations in the TTR gene resulting in TTR variants [2]. Senile Systemic Amyloidosis (SSA) is caused by wild type TTR and is found in the hearts of elderly men, with 25–65% of men aged over 80 years estimated to have SSA [2,3]. The cause of SSA is not known and nor is it known why it occurs predominantly in men [2].

TTR binds many ligands in addition to THs, including antithyroid drugs, plant flavonoids, non-steroidal anti-inflammatory drugs, steroids, salicylates, phenytoin, cardiac agents and analgesics [4,5,6,7,8,9,10,11,12,13,14,15,16,17]. Many of these ligands have strong binding affinities and thus can displace T_4_ binding, which can result in adverse drug responses to clinical treatment regimens [11]. These ligands bind in the central channel of TTR and many have been shown to stabilize the TTR tetramer. Consequently, there has been great interest in developing ligands to stabilize the tetramer to prevent or slow down the rate of progression of TTR amyloid formation [18]. One drug, tafamidis, which binds to the central channel, has been developed for the treatment of TTR type Familial Amyloid Polyneuropathy (TTR-FAP) [13].

**Figure 1:**
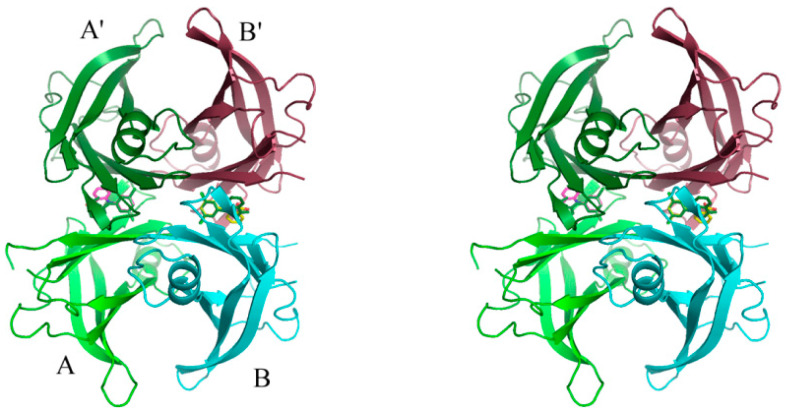
Stereo α-carbon representation of the human transthyretin (hTTR) quaternary structure showing the two independent monomeric subunits A and B (green and cyan) forming the 2-fold related hormone binding site, A′ and B′ (forest and raspberry). The compound of interest, VCP-6, is shown in the binding site with 60% occupancy.

The amino acid sequence of TTRs is highly conserved throughout vertebrate evolution [19]. TTRs are homo-tetramers, assembled from subunits of around 127 residues that have a β-barrel structure formed by two four-stranded β-sheets. The four subunits come together as a dimer of dimers to form a central channel, which contains the TH binding sites [12,20]. Two hormone molecules can bind per tetramer (Figure 1). Although there are two sterically equivalent binding sites in the tetramer, biochemical data indicate that in the general circulation only one site is filled by T_4_. A mechanism of negative cooperativity has been invoked to explain the lesser affinity for the binding of the second hormone to hTTR [21]. Products of thyroxine deiodination have different binding affinities for human TTR (hTTR), with relative values ranging from 100% for 3′,5′,3,5-tetraiodo-L-thyronine (T_4_), 9% for 3′,3,5-triiodo-L-thyronine (T_3_), 680% for 3′,5′,3,5-tetraiodothyroacetic acid (T_4_ac), to less than 0.6% for 3,3′-diiodo-L-thyronine (3,3′-T_2_) [22].

**Figure 2:**
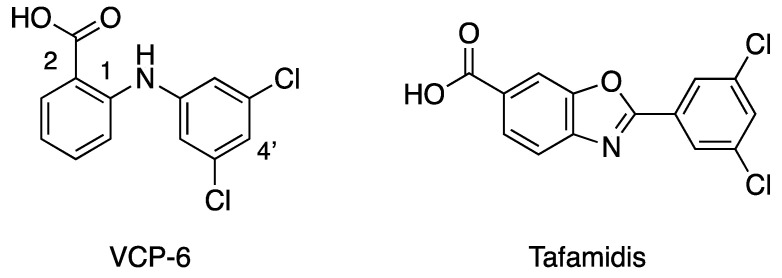
Structures of VCP-6 and tafamidis.

Studies on the binding of analgesic phenylanthranilic acids such as flufenamic, mefanamic or fenclofenac to hTTR show that these compounds are potent competitors of T_4_ binding [4]. Based on these findings, 25 phenylanthranilic acid derivatives were synthesized [9] and were found to be potent competitors for T_4_ binding to hTTR [23]. Of this series, the dichloro derivative VCP-6 (Figure 2) was found to have the greatest affinity, five times that of T_4_. Table 1 shows some examples of the various phenylanthranilic acid ligands that have been investigated and highlights their binding affinities. To investigate the origin of the tight binding of VCP-6, we crystallized it bound to hTTR. Here, we report the structure of VCP-6 in complex with hTTR, analyze the features responsible for VCP-6 binding and compare structures with complexes of thyroxine, tafamidis and 3,3′-diiodo-L-thyronine, a weak binding TH that binds deep in the TTR binding channel.

## 2. Results

**Figure 3:**
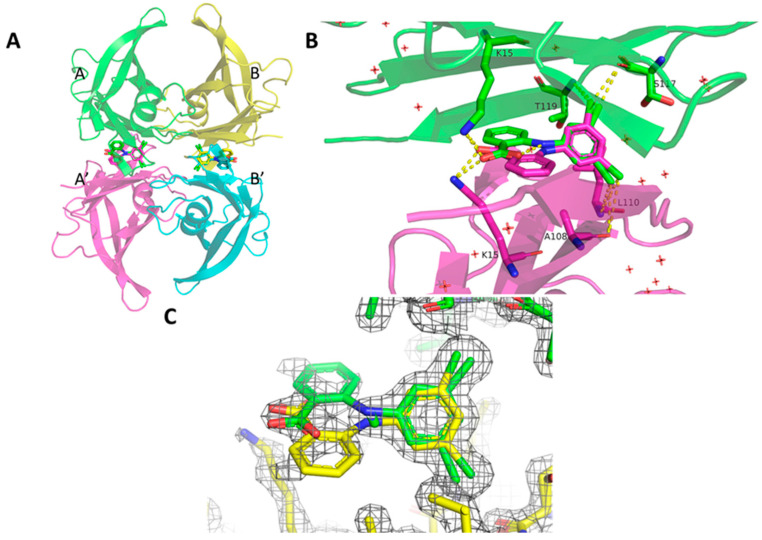
A. The binding mode of VCP-6 (sticks) in the (**A**)-A′ (green and magenta) and (**B**)-B’ (yellow and cyan) binding pockets of the hTTR. VCP-6 binds in the forward binding mode. Note the two alternative positions of the VCP-6 anthranilic acid ring which is supported by the simulated composite omit maps (2fo-fc) maps contoured to 1.3σ shown in panel (**C**). B. Interactions of VCP-6 in the A-A’ dimer. Highlighted via sticks and labelled are the amino acids which directly interact with VCP-6 either via a hydrogen bond (Lys15) or halogen bonds (A108, L110, S117 and T119). The intramolecular hydrogen bond is also shown with yellow dashed lines.

The structure of hTTR in complex with VCP-6 was solved in the I222 space group with one TTR protomer in the asymmetric unit. The physiological TTR tetramer (Figure 3A) was defined using the crystallographic 2-fold axes that runs through the hormone binding central channel. VCP-6 was fitted into the hormone binding pocket (Figure 3C) although there was some lack of density for the 2′-carboxylate ring, however the rest of the density showed clearly. Notably, due to the crystallographic symmetry, VCP-6 fitted into the binding site in two overlapping orientations with an overall binding site occupancy of 60%, with each orientation occupied 30% of the time. The structure was solved to a resolution of 1.52 Å, with model collection and refinement statistics summarized in (Table 2 and Table 3, respectively). Ser17 interacts directly with VCP-6 and displays dual side chain occupancies with 38% occupancy orientating the side chain towards VCP-6 and 62% with it oriented away from the ligand, forming an intramolecular polar interaction with its corresponding Ser117 in the B domain.

In the co-crystallized hTTR complex (Figure 3A,B), VCP-6 binds in the forward mode, with the dichlorophenyl ring bound deep within the channel center. The 3′,5′-chlorine atoms make contacts with four polar atoms in domains A and A’; the backbone carbonyl of Ser117 (O…Cl 3.3/3.2 Å); the backbone carbonyl of Ala108 (O…Cl 3.7/4.2 Å), and the amide nitrogens of Thr119 (N…Cl 3.9/3.9 Å) and Leu110 (N…Cl 3.8/4.0 Å) forming favorable halogen-bonding interactions [24] (Figure 3B).

The 2-carboxylate group is coplanar with the aminophenyl ring (−7^0^) and one oxygen atom forms an intramolecular hydrogen bond with the amine bridge (2.65 Å), which stabilizes the conformation of the diphenylamine ring system, while the other oxygen atom forms an intermolecular hydrogen bond (2.7 Å) with the Nζ of Lys15. These interactions place the 2-carboxyl phenylamine ring coincident with the 2-fold axis in the channel center and locks the molecule in its binding site deep within the channel (Figure 3B).

**Figure 4:**
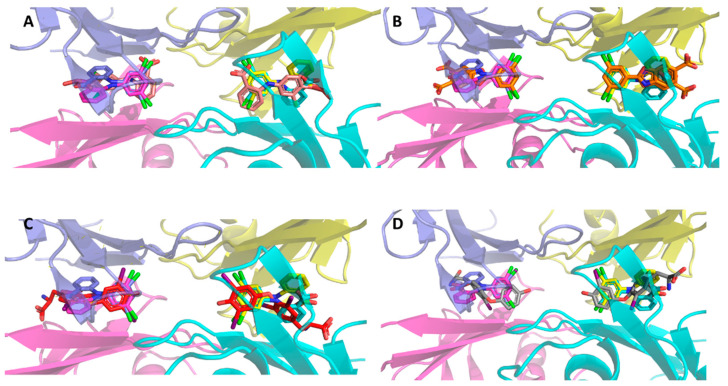
Comparisons of VCP-6 binding with other ligands. VCP-6 is colored as per Figure 3. (**A**) 2-[(3,5-dichloro-4-trioxidanylphenyl)amino]benzoic acid (wheat colored) [25] binds in the reverse mode. The addition of the trioxidane group makes the reverse binding mode favorable, exposing its functional group to the solvent at the channel entrance. (**B**) The drug tafamidis (orange) [13]. Note that the carboxyl ring of tafamidis occupies two alterative positions about the 2-fold axis of the channel. (**C**) Thyroxine (T_4_) (red) [27], showing that VCP-6 binds deeper in the channel than T_4_. (**D**) 3,3′-T_2_ (grey) [7]. Note that the dichlorophenyl ring binds as deeply in the channel as the 3′-iodophenyl ring of 3,3′-T_2_.

The forward binding mode for the hTTR:VCP-6 co-crystallized complex reported here differs from that of a 4′-trioxidaynyl-VCP-6 derivative reported by Wiseman et al. ([25]; PDB 1u21), which binds in the reverse mode that places the 4′-trioxidane functional group (O_3_H) at the channel entrance pointing into the solvent. In the Wiseman structure, the 2-carboxylate group forms intermolecular hydrogen bonds to the hydroxyls of Ser117 and Thr119 (2.2/3.3 Å), and an intramolecular hydrogen bond to the bridging amine (3.0 Å). Comparison of the Wiseman structure with the data reported here shows that the Wiseman ligand is not coincident with the 2-fold axis along the channel and is displaced from the axis (Figure 4A). The chlorine atoms form interactions with the A and A′ side chain methyl groups of Ala108 and with the side chain methylene atoms of Lys15 (3.1/3.5 Å). Forward binding of this derivative would be unfavorable due to steric clashes of the 4-trioxidane group at the tetramer interface. A crystal structure of a VCP-6:hTTR complex formed by soaking crystals of hTTR with ligand has also been reported [26], which shows VCP-6 bound in the reverse mode such that the 2-carboxylic acid bearing aromatic ring occupies the inner halogen binding pocket and forms intermolecular hydrogen bonds with the hydroxyls of Ser117 and Thr119; however, the coordinates of this structure were not deposited, so direct comparison of the binding of VCP-6 cannot be readily made These results are similar to the binding mode observed for a tethered derivative of VCP-6 [25].

## 3. Discussion

Here, we describe the structure of VCP-6 co-crystallized with TTR in the I222 space group. TTR has previously been crystallized in orthorhombic space groups (P2_1_2_1_2 [13,14] and P2_1_22_1_ [28]) and I222 [29,30]. The majority of human transthyretin crystal structures (218 of 240 structures, as of 2022) crystallize in P2_1_2_1_2 [29]. Interestingly, the crystallization condition for the hTTR-VCP complex was based on the condition for the hTTR-tafamidis complex, where the structure was solved in the P2_1_2_1_2 space group. VCP-6 may have a role in altering the condition subtly to promote the I222 space group.

Kinetic stabilization of the TTR tetramer by small molecule competitors of T_4_ binding has been sought as a strategy to prevent or ameliorate the effects of amyloid fibril formation [31]. Structure-based design studies showed that the benzoxazole scaffold produced derivatives that are potent TTR amyloidogenic inhibitors of T_4_ binding to hTTR and that the dichlorophenyl derivative tafamidis selectively binds to TTR and is a potent kinetic stabilizer of both wild type and mutant TTR tetramers [13]. A comparison of the crystal structure of the hTTR:tafamidis complex (Figure 4B) with the VCP-6 complex reveals that tafamidis also binds in a forward mode in the same position along the channel with the dichlorophenyl rings overlapping in the half-occupancy models for refinement. However, unlike VCP-6, the interaction of the carboxylate with Lys15 is much weaker (5.0 Å).

As part of a program to understand the mechanism of molecular recognition for different thyroid hormone metabolites, as well as their binding competitors, we have determined the crystal structures of several TTR-bound complexes [6,7,8,10,12,27,32,33,34,35,36,37]. These data show that unusual binding modes can be accommodated by the protein and that multiple binding orientations are possible for diverse classes of compounds with examples of combinations of “forward”, “reverse” binding orientations and differential displacements (“shuttle”) along the channel axis of the hormone binding site. For example, in the structure of the hTTR-3,3′-T_2_ complex [7], this weak-binding hormone metabolite binds deeper in the ligand channel than does T_4_ [10,32], and it is oriented about 45° relative to that of T_4_. In the case of the bromoflavones, which can exhibit antihormonal properties including inhibition of iodothyronine deiodinase, the synthetic plant flavonoid EMD21388 (3-methyl-4′,6-dihydroxy-3′,5′-dibromoflavone) is the strongest competitor for T_4_ binding to TTR and can alter the circulating total and percentage of free THs and serum thyrotropin concentrations [38,39]. Structures of the hTTR-EMD21388 complex revealed two different binding modes: a forward mode with the dibromophenol ring bound deeper in the channel than T_4_, and a reverse mode with the dibromophenol ring bound toward the channel entrance [6,8,12,35,37]. Comparison of the binding of EMD21388 for hTTR and rat TTR further revealed that the compound binds in two different populations that penetrate 1.6 Å deeper in the rat TTR structure. These data support the role of the 3-methyl of EMD21388 in effective binding and stabilization of the TTR tetramer [37,40].

In the co-crystallized hTTR:VCP-6 complex reported here, VCP-6 is positioned deeper in the channel than T_4_ [27,32] (Figure 4C), and is similar to the binding of the deiodination product, 3,3′-T_2_ [7] (Figure 4D), which has a weak binding affinity (0.6%) [22]. However, unlike 3,3′-T_2_, VCP-6 has the dichlorophenyl ring bound coincident with the 2-fold axis along the channel that permits the chlorine atoms to form strong interactions with the backbone carbonyl atoms (S117 3.5 Å; A108 3.9 Å) with both monomers. Additionally, the formation of an intramolecular hydrogen bond between the 2-carboxylate and the bridging amine of VCP-6 locks the diphenylamine conformation, thereby placing the 2-carboxylate within hydrogen bonding distance to Lys15 Nζ (2.8 Å) in the channel.

Interestingly, VCP-6, a dichloro analog, is slightly more active than corresponding diiodo or dibromo analogs, albeit within 0.5 log units (Table 1). This could be a result of the directionality of the respective halogen bonding interactions, with Cl atoms in halogen bonds known to be more tolerant of angle changes away from linearity [24]. Specifically, the angles range from 101.4° for C-Cl…N (Leu110) to 145.1° for C-Cl…O (Ala108). These somewhat non-linear angles are more tolerated in Cl halogen bonds, compared to Br and I halogen bonds, which tend to have more linear bond angles [24] and could be a reason for why the dichloro derivative is the most potent inhibitor for T_4_ binding (Table 1). An alternative hypothesis could be that the binding pocket is not large enough to accommodate the longer bond lengths of Br or I analogs while optimizing contact with Lys15.

## 4. Materials and Methods

**Expression of hTTR.** Human TTR with a N-terminal hexahistidine tag was expressed in *Escherichia coli* T7 express LysY/I^q^ cells (New England Biolabs) carrying the pRSET_A TTR plasmid (strain JTR_2). JTR_2 cells were grown overnight in 5 mL LB media supplemented with 100 μg/mL ampicillin. The overnight culture was diluted 1:250 into fresh LB media supplemented with 100 μg/mL ampicillin and 10 μg/mL chloramphenicol. The culture was incubated with shaking at 37 °C until OD_600_ = 0.74 and induced with 200 μM isopropyl-β-D-1-thiogalactopyranoside (IPTG). The cells were harvested by centrifugation at 5000× *g* for 20 min, resuspended in START20 buffer (1 × PBS, 350 mM NaCl, pH 7.2, 20 mM imidazole) prior to being frozen at −80 °C.

**Purification of hTTR.** Cell resuspensions of T7 express LysY/I^q^ cells with expressed TTR were thawed and lysed via sonication on ice (5 × 30 s, 40% duty cycle, Branson digital sonifier 450). The lysate was clarified by centrifugation at 40,000× *g* for 1 h, prior to filtration through 0.45 μm and 0.22 μm syringe filters (Merck Millipore) using a disposable syringe. TTR was purified from the lysate via Ni^2+^-IMAC chromatography using Äkta Pure FPLC system (GE Healthcare). The supernatant was loaded onto a 5 mL HisTrap HP (Cytiva Life Sciences) preequilibrated with START20 buffer. The column was washed with 20 column volumes of START20 buffer. The protein was eluted off the column with an imidazole gradient from 20 mM to 500 mM over 12 column volumes. Fractions showing UV absorbance A_280nm_ above baseline were analyzed by SDS-PAGE. Fractions containing protein > 80% pure were pooled, concentrated using a Vivaspin 20 10 kDa MWCO centrifugal concentrator (Sartorius) and loaded onto a Superdex 200 Increase 10/300 GL column (GE Healthcare) preequilibrated with 1×PBS buffer (Gibco). Protein was eluted off the column at 0.7 mL/min. Fractions showing UV absorbance A_280nm_ above baseline were analyzed by SDS-PAGE. Fractions with purity > 99% were pooled and concentrated to 11.54 mg/mL using a Vivaspin 20 10 kDa MWCO centrifugal concentrator (Sartorius).

**Crystallization and Data Collection.** Crystals of hTTR:VCP-6 were grown via sitting-drop vapor diffusion. 1 μL of TTR protein at 11.54 mg/mL was mixed with 1 μL of reservoir solution (1.3 M sodium citrate pH 5.71, 3% glycerol) and 0.1 μL of 100 mM VCP-6 (FocusBio) dissolved in DMSO and equilibrated over a 500 μL reservoir. The crystallization condition used was based on a previously published condition for a TTR-tafamidis crystals [13]. Trays were incubated at room temperature. Crystals grew within 5 days. Crystals were mounted on cryoloops, cryoprotected by passing through Parabar 10312 (Hampton Research) and flash frozen in liquid nitrogen. A 1.52 Å diffraction dataset was collected under cryo conditions at a wavelength of 0.9537 Å using the Eiger X 16M detector on the macromolecular beamline MX2, which is a part of ANSTO [41]. Diffraction data was processed using iMosflm [42] and combined and scaled using AIMLESS [43]. Data collection statistics are summarized in Table 2.

**Structure Solution.** The structure was solved by molecular replacement using Phaser [44] using the structure of hTTR-3,3′-T2 complex (PDB accession 1THA [7]) as the search model. VCP-6 was fitted to the electron density with Phenix.ligandfit [45] with an occupancy of 50%. Refinement was carried out in Phenix.refine [46]. Subsequently, the structure was iteratively rebuilt and refined using Coot [47] and phenix.refine to an R and Rfree of 19.71% and 23.53%, respectively. The occupancy of VCP-6 in the final structure was 30.0%. Refinement statistics are summarized in Table 3. The coordinates for this structure have been deposited with Protein Data Bank (8dw5) and the validation report is available in the Supplementary Materials.

**Table 2:** Data collection statistics for the hTTR-VCP-6 Complex.

Wavelength (Å)	0.9537
Resolution range (Å)	51.06–1.52 (1.55–1.52)
Space group	I 2 2 2
Unit cell dimensions	
a (Å)	41.20
b (Å)	64.08
c (Å)	84.53
α (Å)	90
β (Å)	90
γ (Å)	90
Total reflections	222,685 (11,136)
Unique reflections	17632 (859)
Multiplicity	12.6 (13.0)
Completeness (%)	99.93 (100.0)
Mean I/σ(I)	11.0 (1.0)
Wilson B-factor (Å^2^)	22.79
R_merge_^a^	0.1185 (3.067)
R_meas_^b^	0.1235 (3.19)
R_pim_^c^	0.0346 (0.8693)
CC_1/2_^d^	0.999 (0.337)

The values in parentheses refer to data in the highest resolution shell. ^a^ $R_{merge}=\sum_{hkl}\sum_{i}\left|I_{i}\left(hkl\right)-I\left(hkl\right)\right|\sum_{hkl}\sum_{i}I_{i}\left(hkl\right)$ [48]; ^b^ $R_{meas}=\underset{hkl}{\sum }{\left\{N\left(hkl\right)/\left[N\left(hkl\right)-1\right]\right\}}^{1/2}\times \underset{i}{\sum }\left|I_{i}\left(hkl\right)-I\left(hkl\right)\right|/\underset{hkl}{\sum }\underset{i}{\sum }I_{i}\left(hkl\right)$ [49]; ^c^ $R_{pim}=\underset{hkl}{\sum }{\left\{1/\left[N\left(hkl\right)-1\right]\right\}}^{1/2}\times \underset{i}{\sum }\left|I_{i}\left(hkl\right)-I\left(hkl\right)\right|/\underset{hkl}{\sum }\underset{i}{\sum }I_{i}\left(hkl\right)$ [50]; ^d^ ${\mathrm{CC}}_{1/2}=\sum \left(x-x\right)\left(y-y\right)/{\left[\sum {\left(x-x\right)}^{2}{\left(y-y\right)}^{2}\right]}^{1/2}$ [51].

**Table 3:** Data refinement statistics for hTTR complex.

PDB accession	8dw5
Resolution range (Å)	51.06–1.52 (1.574–1.52)
Reflections used in refinement	17630 (1736)
Reflections used for R-free	1763 (174)
R_work_^a^	0.1971 (0.3369)
R_free_^b^	0.2353 (0.3582)
CC_work_	0.965 (0.641)
CC_free_	0.923 (0.657)
Number of non-hydrogen atoms	957
macromolecules	902
ligands	18
solvent	37
Protein residues	116
RMS (bonds)	0.014
RMS (angles)	1.66
Ramachandran favored (%)	98.25
Ramachandran allowed (%)	0.88
Ramachandran outliers (%)	0.88
Rotamer outliers (%)	1.02
Clashscore	6.09
Average B-factor (Å^2^)	30.57
Macromolecules (Å^2^)	30.51
Ligands (Å^2^)	22.80
Solvent (Å^2^)	35.71
Number of TLS groups	8

Statistics for the highest-resolution shell are shown in parentheses. ^a^ $R_{work}=\sum |F_{o}-F_{c}|/\sum \left|F_{o}\right|$ for all data with $F_{o}>2\sigma \;\left(F_{o}\right)$, excluding data to calculate $R_{free.}$ ^b^ R_free_ = $\sum |F_{o}-F_{c}|/\sum \left|F_{o}\right|$ for all data with $F_{o}>2\sigma \;\left(F_{o}\right)$, calculated with 10% of reflections that are randomly chosen [52].

## 5. Conclusions

In this study, we present the structure of the phenylanthranilic acid VCP-6, bound to hTTR. VCP-6 binds TTR in the forward mode, with the dichlorophenyl ring bound deep within the channel center. Two symmetry-related and overlapping bound poses are present. The 3′,5′-chlorine atoms make halogen-bonding interactions with the halogen binding pockets created by residues Ala108, Leu110, Ser117 and Thr119 in each protein monomer. The 2-carboxylate group is coplanar with the aminophenyl ring and makes an intramolecular hydrogen bond with the bridging amine that stabilizes the conformation of the diphenylamine ring system. The other carboxylate oxygen atom makes an intermolecular hydrogen bond with the amino group of Lys15. This location of the dichlorophenyl ring is very similar to that observed for the dichlorophenyl ring of tafimidis. This crystal structure provides a structural basis for the clinically observed interactions of similar phenylanthranilic acids (e.g., flufenamic acid) with TTR [4] and provides a template for the design of TTR stabilizers for the prevention of TTR amyloidosis.

## Figures and Tables

**Table 1:** Affinities of a selection of phenylanthranilic acid derivatives for hTTR ^a^.

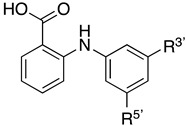			
Compound	R3′	R5′	pIC_50_
T_4_	-	-	6.43
VCP-2	Cl	H	6.9
VCP-5	CF_3_	CF_3_	7.09
VCP-6	Cl	Cl	7.14
VCP-16	I	I	6.89
VCP-17	Br	Br	6.73
VCP-19	Br	H	6.80
VCP-20	I	H	7.07
VCP-21	CH_3_	H	6.32
VCP-25	CN	H	6.24
Flufenamic acid	CF_3_	H	6.78

^a^ Data taken from Chalmers D, Ph.D. thesis, University of Melbourne, 1993 [23] using the method described by Munro et al. [4].

## Data Availability

Coordinates of the complex are deposited (PDB ID 8dw5).

## Supplementary Materials

The following are available online at https://www.mdpi.com/article/10.3390/molecules27217206/s1, Full wwPDB X-ray Structure Validation Report.
